# Adolescents Admitted for Suicide Attempts to a Tertiary Pediatric Hospital in Romania: An Eleven-Year Retrospective Study

**DOI:** 10.3390/children13040519

**Published:** 2026-04-08

**Authors:** Andreea Lescaie, Gabriela Viorela Nitescu, Andreea Catalina Stratula, Dora Andreea Boghițoiu, Cristina Iolanda Vivisenco, Andreea Manolache, Diana Georgiana Cotuna, Alina Mitrea, Florina Rad

**Affiliations:** 1 Discipline of Pediatrics, Carol Davila University of Medicine and Pharmacy, 030167 Bucharest, Romania; andreea.lescaie@drd.umfcd.ro (A.L.); andreea-catalina.stratula@drd.umfcd.ro (A.C.S.); dora.boghitoiu@umfcd.ro (D.A.B.); cristina.vivisenco@umfcd.ro (C.I.V.); 2 Pediatric Poison Center, Grigore Alexandrescu Clinical Emergency Hospital for Children, 011743 Bucharest, Romania; andreeamanolache@yahoo.com; 3 Pediatric Psychiatry Outpatient Department, Grigore Alexandrescu Clinical Emergency Hospital for Children, 022561 Bucharest, Romania; cotuna.diana@yahoo.com; 4 Department of Psychiatric Specialties, Geneva University Hospitals, 1205 Geneve, Switzerland; alina.mitrea@hug.ch; 5 Child and Adolescent Psychiatry Department, Carol Davila University of Medicine and Pharmacy, 030167 Bucharest, Romania; florina.rad@umfcd.ro; 6 Child and Adolescent Psychiatry Department, Prof. Dr. Al Obregia Clinical Hospital of Psychiatry, 041914 Bucharest, Romania

**Keywords:** suicide, suicide attempts, adolescent, poisoning, mental health

## Abstract

**Highlights:**

**What are the main findings?**

- Suicide attempts among adolescents increased significantly over an 11-year period.
- Suicide attempts showed sex- and method-specific patterns.

**What are the implications of the main findings?**

- Prevention efforts should be sex-specific, focusing on early mental health screening.
- The integration of toxicological and psychiatric care is crucial for the effective management of adolescents who have attempted suicide.

**Abstract:**

**Introduction:** Adolescent suicide behavior is a global concern and a leading cause of morbidity and mortality in this age group. Pharmaceutical ingestion is a frequent method of suicide attempts, requiring toxicology and psychiatric interventions. This study analyzed data from a pediatric tertiary hospital to elucidate the trends, demographics, and methods used. **Methods**: This retrospective study was conducted at a single tertiary pediatric hospital in Romania and included adolescents aged 10–18 years admitted for suicide attempts between 2014 and 2024. Data extracted from electronic medical records included age, sex, residence, suicide method, psychiatric history, and clinical outcomes. Temporal trends were analyzed using regression-based methods, and group comparisons were performed using chi-square tests, with statistical significance set at *p* < 0.05. **Results**: The study included 1840 adolescents, with a significant increasing trend over time (*p* < 0.0001), but with a transient decline in 2020. The female-to-male ratio was 5.4:1 (*p* < 0.0001). The median age of the patients was 15.2 years. The suicide attempt methods were pharmaceutical poisoning (95%), chemical ingestion (4%), and violent methods (1%). Females were more prone to pharmaceutical poisoning, while males were associated with chemical ingestion and violent methods (*p* < 0.0001). Previously documented psychiatric disorders were present in 32.8% of patients, while 9.6% had a history of prior suicide attempts. Two fatalities (0.1%) were recorded, both involving defenestration. **Conclusions**: Hospital-treated suicide attempts among Romanian adolescents increased over time, mainly affecting females through pharmaceutical poisoning. Adolescents without prior psychiatric diagnoses reveal gaps in early identification and prevention. These findings highlight important patterns among hospital-treated adolescents and may inform targeted prevention efforts.

## 1. Introduction

Suicide attempts represent a major global health challenge, particularly among adolescents and young adults. The World Health Organization (WHO) reports that suicide is the second leading cause of death globally in the 15–24-year age group, with approximately 700,000 annual deaths [1]. Suicide attempts, a crucial indicator of future risk, affect an estimated 10–20% of adolescents worldwide, with variations across regions and cultures [2,3].

Adolescent suicidal behavior is shaped by a complex interplay of individual, family, and social determinants [4,5]. In hospital-treated adolescent populations, pharmaceutical self-poisoning is consistently reported as the most common method of suicide attempts, particularly in high-income and middle-income settings, and requires specialized multidisciplinary management [6,7].

Globally, the epidemiology of adolescent suicidal behavior shows substantial regional variations. Population-based studies and international surveillance systems indicate declining or stabilizing suicide mortality trends in several European countries, while increasing rates of suicide attempts and self-harm presentations have been reported in parts of North America, particularly among adolescent girls [8,9,10,11]. In contrast, several Asian and low- and middle-income regions continue to experience higher suicide mortality rates, often influenced by socioeconomic stressors, access to lethal means, and disparities in mental health care [12,13]. Across regions, non-fatal suicide attempts are far more frequent than completed suicides, with marked differences in sex distribution and preferred methods [14]. These heterogeneous global patterns underscore the importance of region-specific epidemiological data to inform targeted prevention strategies.

Evidence also indicates a rise in adolescent suicide attempts across Eastern European countries over the past decade, especially in females and involving self-poisoning; however, published data remain limited and originate mainly from settings in Poland and Turkey [15,16,17].

National registries and surveillance systems play a critical role in monitoring suicide attempts, identifying population-level risk factors, and informing evidence-based prevention strategies. Several high-income countries, such as Denmark, Sweden, Taiwan, Japan, Uruguay, and Spain, have established national registries that track suicide attempts [6,18,19]. These systems enable longitudinal epidemiological research and near real-time surveillance by primarily capturing hospital-treated cases.

The WHO’s Mental Health Atlas country profile indicates that Romania lacks a dedicated suicide attempt reporting and prevention strategy, underscoring the importance of strengthening surveillance and integrating mental health assessment pathways within pediatric care settings [20,21].

Currently, national surveillance in Romania relies on mortality statistics and lacks systematic reporting of non-fatal suicide attempts among adolescents [20,21]. As a result, hospital-treated suicide attempts in adolescents are severely underreported and largely confined to reports from a pediatric psychiatry unit, which documented increasing admissions for self-poisoning, particularly in girls after the COVID-19 pandemic period [22,23,24]. There are no reports of hospital-treated suicide attempts in adolescents that analyzed method-specific patterns, sex differences, and long-term temporal trends.

Beyond epidemiological description, hospital-based data on suicide attempts provide critical insight for prevention and health-system planning, as non-fatal suicide attempts represent the strongest predictor of future suicide [25]. In settings where national registries of suicide attempts are lacking or incomplete, hospital-based surveillance plays an essential role in identifying temporal trends, method distribution, and service needs, thereby informing targeted prevention strategies and the integration of multidisciplinary management [26].

This study aimed to fill this gap by analyzing 11 years (2014–2024) of data from one of the largest tertiary pediatric hospitals in Romania. Specifically, the objectives were to describe demographic patterns, suicide methods, temporal trends, and psychiatric comorbidities. By situating these clinical data alongside global epidemiological findings, this study aims to provide context-specific evidence to inform future prevention strategies and integrated multidisciplinary care pathways in Romania.

## 2. Materials and Methods

### 2.1. Study Design and Setting

This retrospective epidemiological study analyzed adolescents admitted for suicide attempts to the Grigore Alexandrescu Clinical Emergency Hospital for Children in Bucharest, Romania.

This tertiary pediatric hospital serves as a principal multidisciplinary institution, addressing all types of medical and surgical emergencies occurring in the southern and southeastern regions of the country. The hospital comprises several specialized units, including a pediatric poison center and a pediatric polytrauma center. Furthermore, it functions as a referral center for complex cases that cannot be managed by regional hospitals nationwide. Consequently, the study captured a large and clinically relevant subset of adolescents presenting with suicide attempts that required hospital admission and multidisciplinary management within tertiary care settings.

All adolescents aged 10–18 years who were admitted for suicide attempts between 1 January 2014 and 31 December 2024 were included, irrespective of the suicide method. The exclusion criteria were age outside the 10–18 years range, non-suicidal self-injury (NSSI) cases, cases not admitted to the hospital, and follow-up admissions for sequelae of a previous suicide attempt. Suicide-related deaths that occurred at home and were subsequently handled by forensic medicine services have been excluded from this analysis.

The age range of 10–18 years for the included cases was selected in accordance with the WHO’s definition of adolescence (10–19 years) and the pediatric scope of care of the study hospital, as pediatric hospitals routinely manage patients up to 18 years of age [27].

Suicide attempts were defined as acts of intentional self-harm involving methods associated with a risk of death and were clinically recorded as suicide attempts (fatal or non-fatal) in hospital medical records.

### 2.2. Data Extractions and Classifications

Data were retrospectively extracted from the hospital’s electronic medical records into a spreadsheet using predefined variables. The collected variables included age, sex, urban or rural residence, suicide method, relevant medical and psychiatric history, and clinical outcomes. The selection of variables was constrained by the retrospective nature of the study and the structure of the routinely collected medical records. Standardized socioeconomic indicators, therefore, could not be reliably included as analytic variables. All records in the spreadsheet were reviewed for completeness and internal consistency prior to the statistical analysis.

Information on precipitating factors and contextual circumstances (e.g., family conflict, romantic relationship issues, academic stress, and bullying) was extracted from unstructured clinical documentation when explicitly recorded in medical files. These factors were identified based on patient self-report during clinical interviews, caregiver accounts, and clinician narrative notes documented during emergency, pediatric, psychological, or psychiatric evaluations.

Suicide attempts were identified based on detailed clinical assessment documented in the medical records at the time of hospital admission and relied on routine institutional practice involving multidisciplinary clinical judgment by pediatricians, with psychological and psychiatric evaluations performed when medically feasible. Although standardized psychometric instruments were not systematically applied throughout the study period, assessments were guided by clinical criteria consistent with international definitions of suicide attempts, including patient history, circumstances of the act, reported intent, lethality expectation, and collateral information from caregivers. This consistent institutional approach was maintained throughout the study period and ensured diagnostic coherence in differentiating between suicide attempts and NSSI, while reflecting real-world clinical practice in a tertiary pediatric hospital setting.

The cases were classified by suicide attempt methods into pharmaceutical poisoning, chemical substance poisoning, and violent methods.

Pharmaceutical poisoning included cases of adolescents who ingested prescription or over-the-counter medications. Chemical substance poisoning included cases of adolescents who ingested non-pharmaceutical chemicals.

Violent methods included all other types of suicide attempts apart from poisonings; however, this dataset identified only defenestration and hanging cases. No cases of suicide attempts involving falling under vehicles or self-inflicted gunshot wounds were identified during the study period.

Based on multidisciplinary clinical assessments by pediatricians, clinical psychologists, and pediatric psychiatrists, all cases involving isolated self-cutting were categorized as NSSI and excluded from the analysis. However, cases in which self-cutting occurred in combination with a primary suicidal act were included and classified according to the principal method of the suicide attempt.

### 2.3. Statistical Analysis

All statistical analyses were performed using XLSTAT software, version 2025.27.1, (Addinsoft, France). Descriptive statistics were used to summarize demographic and clinical characteristics of the study population. Continuous variables are presented as medians, and categorical variables are reported as absolute numbers and percentages of the total.

Overall temporal trends in annual counts of suicide attempts were assessed using Poisson regression with calendar year entered as a continuous predictor; results are summarized as incidence rate ratios (IRRs) with 95% confidence intervals and *p*-values. To evaluate whether specific calendar years deviated from this underlying linear trend, we refitted the Poisson model with an additional binary indicator for the year of interest (e.g., 2020 or 2022) and compared models with and without this term using likelihood ratio χ^2^ tests. Changes in proportions over time (e.g., sex-specific temporal trends) were evaluated using the Cochran–Armitage test for trend.

Associations between sex and suicide attempt methods were analyzed using Pearson’s χ^2^ test of independence. Odds ratios (ORs) and 95% confidence intervals (95% CIs) were calculated to quantify the strength and direction of the associations. The overall effect size for the relationship between sex and method of suicide attempt was assessed using Cramér’s V.

Statistical significance was set at *p* < 0.05 for all analyses.

Multivariable modeling was not performed, as the primary aim of this study was descriptive epidemiological characterization. The set of available covariates was limited to routinely documented demographic and clinical variables, while key psychosocial and socioeconomic factors were not consistently reported for reliable adjustment. In this context, univariable and stratified analyses were considered appropriate to avoid over-interpretation and residual confounding that could arise from incomplete or inconsistently recorded variables. The study was not designed to assess independent risk factors or causal associations, and all analyses were interpreted accordingly.

## 3. Results

### 3.1. Demographic Characteristics

The study cohort included 1840 adolescents admitted for suicide attempts between 2014 and 2024.

**Figure 1:**
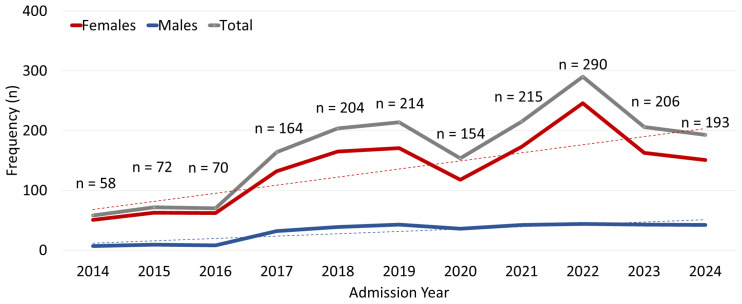
Annual distribution of suicide attempts by sex among adolescents during the study period.

Over the period examined, the annual number of attempts exhibited a statistically significant upward trend in the Poisson regression model (IRR per year = 1.10, 95% CI 1.08–1.12, *p* < 0.0001, Figure 1). When we added a binary indicator for 2020, the model indicated a significantly lower rate than expected from the underlying trend (IRR = 0.82, 95% CI 0.69–0.97, likelihood ratio χ^2^ = 5.53, *p* = 0.019), coinciding with the onset of the COVID-19 pandemic. In contrast, the inclusion of a 2022 indicator showed a significantly higher rate than expected (IRR = 1.44, 95% CI 1.26–1.64, likelihood ratio χ^2^ = 13.3, *p* < 0.001), corresponding to a peak incidence in 2022. After the 2022 peak, case numbers for 2023 and 2024 appeared to stabilize without evidence of a sustained decline.

Females constituted a substantial majority, representing 84.4% (*n* = 1553) of the cases, whereas males accounted for only 15.6% (*n* = 287) of the cases. The resulting female-to-male ratio of 5.4:1 was statistically significant compared to an equal distribution (χ^2^ = 867.4, *p* < 0.0001), indicating a pronounced sex disparity. The yearly proportions of female-to-male cases showed evidence of a linear change over time, indicating that both sexes increased proportionately over the study period (χ^2^ = 0.034, *p* = 0.85; Figure 1).

Analysis of residential status revealed that 55% (*n* = 1012) of the cases resided in urban areas, whereas the remaining 45% (*n* = 828) resided in rural areas. This slight urban predominance was stable throughout the study period.

**Figure 2:**
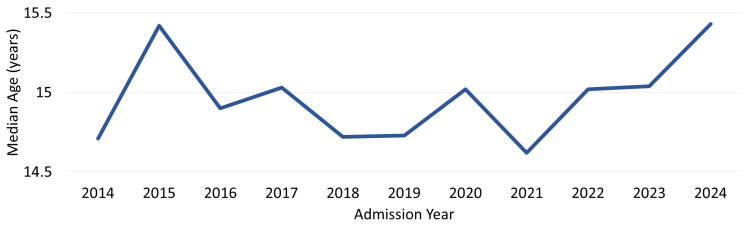
Median age of adolescents with suicide attempts by admission year.

The median age of adolescents at the time of the suicide attempt was 15.2 years, with annual medians ranging between 14.6 and 15.4 years. No statistically significant variation in the median age was observed across the years studied (*p* = 0.53, Figure 2).

### 3.2. Methods and Context of Suicide Attempts

**Figure 3:**
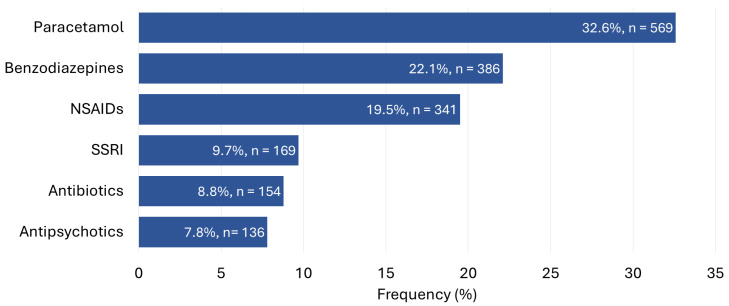
Pharmaceutical substances involved in adolescent suicide attempts, limited to medications identified with a frequency greater than 5% of cases. Percentages may exceed 100% because some adolescents ingested more than one pharmaceutical substance. Abbreviations: NSAIDs, nonsteroidal anti-inflammatory drugs; SSRI, selective serotonin reuptake inhibitor.

Pharmaceutical poisoning was the most common method of suicide attempt in this cohort, accounting for 95% (*n* = 1748) of all cases. Although its high prevalence remained consistent across all years, the annual number of suicide attempts involving pharmaceuticals increased significantly, paralleling the overall increase in the number of cases. The most commonly ingested drug in cases of pharmaceutical poisoning was paracetamol (32.6%, *n* = 569), followed by benzodiazepines (22.1%, *n* = 386) and nonsteroidal anti-inflammatory drugs (19.5%, *n* = 341) (Figure 3).

Approximately 52.3% (*n* = 914) of pharmaceutical poisoning cases involved the ingestion of a single pharmaceutical substance, whereas the others involved multiple substances ranging from two to ten medications. The ingestion of multiple pharmaceuticals was more frequent in males than in females. The pharmaceuticals used for suicide attempts were already available at home in 82.7% (*n* = 1445) of the cases, whereas in the remaining 17.3% (*n* = 303) of the cases, adolescents procured them from friends or drug stores.

Chemical substance ingestion, involving non-pharmaceutical toxic agents such as detergents, caustic substances, or pesticides, comprised 4% (*n* = 74) of all attempts.

Violent methods, including defenestration and hanging, were comparatively rare, representing only 1% (*n* = 18) of the cases.

The distribution of suicide methods should be interpreted in the context of the study setting, as the hospital functions as a tertiary referral center for severe pediatric emergencies, including both toxicological and traumatic cases, which may influence the case mix of admitted patients.

**Figure 4:**
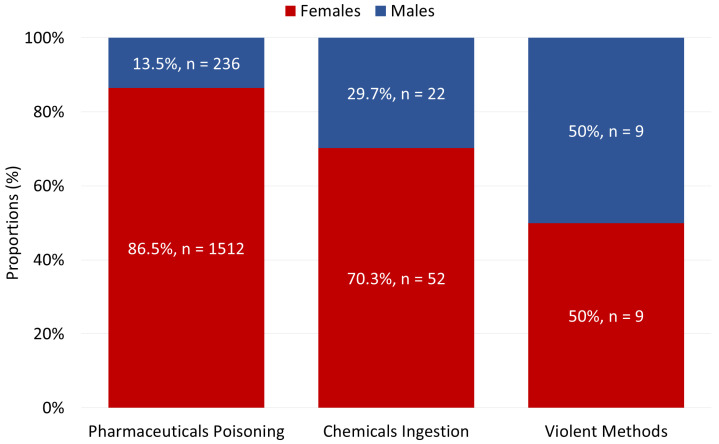
Sex-specific distribution of suicide attempt methods among adolescents.

Statistically significant sex differences were observed in the distribution of suicide attempt methods (χ^2^ = 33.53, *p* < 0.0001, Figure 4), but the magnitude of this association was small (Cramér’s V = 0.14). Females predominantly used pharmaceutical poisoning (χ^2^ = 27.13, *p* < 0.0001; OR = 3.27, 95% CI: 1.77–6.06), whereas males were more likely to engage in chemical substance ingestion (χ^2^ = 14.08, *p* < 0.0001; OR = 2.73, 95% CI: 1.57–4.78) and especially in violent suicide attempts (χ^2^ = 16.93, *p* < 0.0001; OR = 6.41, 95% CI: 2.50–16.40).

Most suicide attempts (78%, *n* = 1435) occurred in the adolescents’ homes. The remaining cases occurred at friends’ homes or in public spaces.

Family conflict was the predominant precipitant, accounting for approximately 28.6% (*n* = 526) of the cases. Romantic conflicts were implicated in approximately 11.3% (*n* = 208) of the suicide attempts. Additional triggers identified in a smaller number of cases included academic failure (0.9%, *n* = 16), bullying (0.8%, *n* = 15), sexual violence (0.3%, *n* = 6), and grief reactions (0.3%, *n* = 5). The remaining cases lacked specific documented triggers for the suicide attempts. Severe family dysfunction was identified in 6.6% (*n* = 122) of cases.

### 3.3. Psychiatric Comorbidities Prior to Suicide Attempt

Psychiatric disorders were previously documented in the patient’s medical history in 32.8% (*n* = 603) of the cases, most of them being females (*n* = 575, 95.4%). Among those with known psychiatric diagnoses, 8.3% (*n* = 49) had not received prior pharmacological psychiatric treatment and were managed solely with psychotherapy. These diagnoses reflect psychiatric conditions recorded in the medical history prior to the index suicide attempt, without systematic classification by diagnostic category.

A subset of patients (9.6%, *n* = 176) had at least one prior medically documented suicide attempt. All previous attempts involved intentional poisoning with pharmaceuticals. Except for two male patients with a history of prior suicide attempts, all other patients were female.

Substance use disorder was present in 3.5% (*n* = 65) of the adolescents included in this study, most of whom were males (*n* = 58, 89.2%).

### 3.4. Multidisciplinary Team Involvement

All adolescents included in this study were initially evaluated in the emergency department. However, this study did not document the number of individuals who presented directly or were referred from other medical facilities.

Admission to the intensive care unit (ICU) was required in 4.6% (*n* = 84) of the cases. Stratified by the method of suicide attempt, ICU care was required for 63 of 1748 patients with pharmaceutical poisoning, 3 of 74 patients with chemical poisoning, and all patients with violent methods of suicide attempts. The sex-distribution of patients required ICU showed a female-to-male ratio of 4.6:1.

Cases involving suicide attempts through poisoning, whether by pharmaceuticals or chemicals, were admitted to the pediatric poison center, where they received medical management from clinical toxicologists and pediatricians. In contrast, cases involving violent suicide attempts were managed by trauma specialists in surgical departments.

An initial psychological evaluation was conducted as soon as medically feasible in 78.3% (*n* = 1441) of the cases. Furthermore, 20.4% (*n* = 375) of the patients received a psychiatric evaluation during hospitalization in either a medical or surgical unit.

Based on the severity of their mental health status, 7% (*n* = 128) of the adolescents were transferred to a pediatric psychiatry unit following the resolution of somatic symptoms and complications. The sex distribution of patients that required transfer to a psychiatric unit matched the overall cohort, with a female-to-male ratio of 5.1:1. The remaining cases were referred to a pediatric psychiatry outpatient department after discharge.

### 3.5. Clinical Severity and Outcomes

In most cases (67.7%, *n* = 1246), adolescents exhibited minor symptoms associated with suicide attempts, all of which involved poisoning, either pharmaceutical or chemical.

Complications were observed in 10.4% (*n* = 192) of the cases. Among these, most individuals achieved complete physical recovery with no sequelae one-year post-attempt. However, three cases (two females and one male) involving defenestration resulted in orthopedic sequelae, necessitating referral to a medical rehabilitation clinic.

Fatalities were recorded in only two male cases (0.1%), both resulting from defenestration from a height exceeding 10 m. No fatalities were attributed to pharmaceutical or chemical poisoning.

## 4. Discussion

This retrospective study provides eleven-year data of hospital-treated suicide attempts among adolescents. Although the results cannot be generalized to the overall adolescent population, this study provides a comprehensive account of adolescent suicide attempts managed within a hospital setting. When interpreted with international literature, the findings highlight shared patterns and specific features relevant to adolescent suicidality.

### 4.1. Temporal Trends and the Impact of COVID-19

Previous studies have reported substantial international variability in the prevalence of adolescent suicide attempts, with lifetime estimates ranging from approximately 7% in some Asian countries to over 60% in Pacific regions, such as Samoa [28,29]. In high-income regions, including Europe and the United States, prevalence estimates are generally lower, most commonly between 4% and 12%; however, a sustained increase in adolescent suicide attempts has been documented since the late 2000s [8,30,31,32,33]. Despite the implementation of preventive initiatives and increased public awareness, suicidal behavior among adolescents continues to represent a growing public health concern [28,34].

The present study identified a statistically significant increase in hospital-treated suicide attempts among adolescents admitted to a tertiary pediatric referral center in Romania over the 2014–2024 period, with a peak observed during the second year of the COVID-19 pandemic. These findings are consistent with reports from other European countries and North America, where rising trends in adolescent suicide attempts have been linked to an increasing burden of mental health disorders, persistent psychosocial stressors, and changes in social and digital environments [35,36,37]. The alignment of this study data with international trends suggests that patterns of hospital-treated adolescent suicidality observed in this setting may be influenced by similar global determinants, despite differences in healthcare organization, socioeconomic conditions, and cultural context.

The COVID-19 pandemic was associated with notable temporal variations in hospital-treated suicide attempts in this cohort. A transient decline in admissions was observed in 2020, coinciding with the onset of pandemic-related restrictions, followed by a rebound and peak incidence in 2022. International evidence regarding the impact of the pandemic on adolescent suicidal behavior has been heterogeneous, with several studies reporting increased emergency department presentations for suicide attempts, particularly among adolescent girls [10,38]. In contrast, the initial decline observed in the present study may be explained by several factors, including reduced healthcare-seeking behavior, fear of hospital exposure, restricted mobility during lockdowns, and changes in referral patterns, rather than a true reduction in suicidal behavior [39,40,41].

The subsequent rebound and peak in 2022 may reflect a delayed and cumulative effect of prolonged pandemic-related stressors on adolescent mental health, including social isolation, educational disruption, family stress, and reduced access to support services. Similar delayed increases in pediatric morbidity following the pandemic have been reported both internationally and in Romania, supporting the hypothesis of a broader post-pandemic surge in healthcare utilization and mental health crises among children and adolescents [42,43].

During the 2023–2024 period, the number of cases appeared to stabilize or decline modestly compared to the post-pandemic peak. However, this pattern should be interpreted cautiously, as a longer follow-up is required to determine whether it represents a sustained shift or a transient fluctuation.

It should be emphasized that the observed temporal changes are based on hospital-treated cases, and interpretations regarding underlying mechanisms remain hypothetical. Overall, the “dip-and-rebound” trajectory observed in this cohort underscores the importance of continuous surveillance and sustained mental health interventions, as short-term reductions in hospital presentations during public health crises may obscure ongoing or emerging risks among adolescents who would otherwise present for hospital care.

### 4.2. Methods of Suicide Attempts

The present study demonstrates a clear predominance of pharmaceutical poisoning as the method of suicide attempts among adolescents, consistent with international evidence identifying self-poisoning as the most common method of suicide attempts in this age group, particularly in high-income and middle-income countries [30,32,38]. This pattern reflects the influence of method availability, as medications are widely accessible within the home environment and are frequently used in impulsive suicidal acts.

The substances most commonly involved (paracetamol, benzodiazepines, and nonsteroidal anti-inflammatory drugs) mirror those reported in other pediatric cohorts, where over-the-counter and commonly prescribed medications are disproportionately represented [14,44,45]. From a preventive perspective, these findings highlight the importance of safe medication storage and prescription oversight.

Despite the high prevalence of pharmaceutical and chemical poisoning, no fatalities related to these methods were recorded in this cohort. This observation aligns with previous studies showing that self-poisoning is generally associated with lower lethality compared with violent methods, and that non-fatal attempts substantially outnumber completed suicides in adolescence [30,32,38].

In contrast, violent methods accounted for a small proportion of cases but were associated with greater clinical severity than other methods. Violent methods, including defenestration and hanging, accounted for all recorded fatalities, consistent with international evidence of their higher lethality [46,47,48]. Firearm-related suicide attempts, frequently reported among male adolescents in other settings [45], were not identified in this study, likely reflecting Romania’s restrictive firearm legislation and limited access.

Comprehensive characterization of suicide attempt methods is crucial for informing prevention strategies, optimizing clinical responses, and identifying modifiable risk factors beyond those identified in mortality-based studies. It is important to note, however, that this dataset may underestimate the prevalence of violent methods due to the exclusion of cases that did not survive to be admitted to the hospital.

### 4.3. The “Gender Paradox”

A key finding of this study is the marked predominance of females among adolescents presenting with suicide attempts, with a female-to-male ratio exceeding those commonly reported in the international literature (typically 2:1 to 4:1) [48,49,50]. However, the practical significance of this difference should be interpreted in the context of a hospital-based sample predominantly capturing non-fatal cases.

Females predominantly used pharmaceutical poisoning, a method associated with lower lethality, whereas males were more likely to engage in chemical ingestion, multiple-substance overdoses, or violent methods [28,29,30,31,32]. This distribution aligns with the well-described “gender paradox” in suicide, whereby females exhibit higher rates of suicide attempts, while males account for the majority of suicide deaths [51,52].

Hospital-based data may further accentuate this pattern by preferentially capturing non-fatal attempts, which are more frequent among females, while underrepresenting fatal events occurring outside clinical settings, thereby potentially exaggerating female predominance relative to the broader epidemiology of suicidal behavior. In the present study, the observed sex differences were statistically significant but were associated with small effect sizes, indicating limited practical magnitude.

These sex-specific differences underscore the importance of tailored prevention strategies that account for distinct risk profiles, method availability, and help-seeking behaviors [53,54].

### 4.4. Triggers and Risk Factors

Consistent with previous research, psychiatric disorders represent an important risk factor for suicide attempts during adolescence [55]. However, in the present cohort, approximately two-thirds of adolescents had no previously documented psychiatric diagnosis at the time of the suicide attempt. This finding suggests that suicidal behavior often represents the first clinically recognized manifestation of underlying mental health difficulties, rather than occurring in the context of an established psychiatric history, as also reported in other studies [56,57]. These data highlight potential gaps in the early identification and access to mental health care among adolescents presenting with suicide attempts in hospital settings.

Family conflict was the most frequently documented precipitating factor, and most suicide attempts occurred within the home environment. Similar family- and interpersonal-related stressors have been reported in international studies [58,59,60]. In contrast, academic stress and bullying were infrequently documented, despite being well-established risk factors in prior research [61,62]. However, this finding should be interpreted cautiously, as it likely reflects documentation practices in routine medical records rather than the true prevalence of psychosocial triggers.

Similarly, the presence of NSSI, a strong predictor of suicide attempts with a high reported prevalence in adolescent populations [60,63], was not systematically captured in this study, representing an important limitation of the retrospective design and further restricting the interpretation of psychosocial triggers.

Substance use disorder was identified in a minority of cases, consistent with previous findings that substance use may function as both a proximal trigger and a marker of broader psychosocial vulnerability [64].

Emerging contextual factors, including social media exposure, cyberbullying, and sedentary behavior, have been increasingly associated with adolescent suicidal behavior [65,66,67,68,69] but could not be reliably assessed within the scope of this study. Together, these findings highlight the multifactorial nature of adolescent suicidality, while also underscoring the limitations of routine hospital documentation in capturing psychosocial risk factors and the need for a standardized assessment of psychosocial risk factors in both clinical practice and future research.

### 4.5. Strengths, Limitations, and Future Directions

This study has several strengths that enhance its contribution to the literature on adolescent suicidal behaviors. The extended eleven-year observation period, encompassing both the pre-pandemic and post-pandemic phases, allowed for the assessment of long-term temporal trends and pandemic-related variations. In addition, the large sample drawn from a tertiary pediatric hospital provides insights into clinically significant, hospital-treated suicide attempts. The detailed characterization of demographic data and suicide methods further strengthens the relevance of the findings, particularly given the scarcity of long-term data from Eastern European settings.

This study has several limitations. The single-center design may limit generalizability to other regions or healthcare systems. As only hospital-admitted adolescents were included, less severe attempts, as well as suicide deaths occurring before hospital presentation, were not captured, likely resulting in an underestimation of the true community burden.

The retrospective design and reliance on routine medical records constitute further limitations. Psychosocial variables such as bullying, academic stress, socioeconomic factors, substance use, and non-suicidal self-injury were inconsistently documented, and standardized psychometric instruments were not systematically applied. Emerging risk factors, such as social media exposure and cyberbullying, could not be reliably assessed.

Future research should address these limitations using multicenter and prospective designs that integrate data from emergency departments, psychiatric services, schools, and community settings. The development of standardized data collection tools and national surveillance systems would facilitate more comprehensive monitoring of suicide attempts and enable the evaluation of prevention strategies over time. Further studies should also explore the mechanisms underlying recent changes in suicide attempts, with particular attention to post-pandemic effects, digital media exposure, and high-risk subgroups. Such efforts are essential for informing targeted, evidence-based interventions and improving outcomes for adolescents at risk of suicidal behavior.

## 5. Conclusions

This study provides long-term, hospital-based evidence on adolescent suicide attempts treated at a tertiary pediatric hospital in Romania. Over an eleven-year period, increasing numbers of hospital-treated cases were documented, with marked sex difference, a predominance of pharmaceutical self-poisoning, and substantial multidisciplinary care needs. The fact that most adolescents had no previously documented psychiatric diagnoses highlights potential gaps in early identification and prevention.

Given the complexity and urgency of this public health challenge, integrated and multidisciplinary strategies are essential. The establishment of national surveillance systems to monitor adolescent suicide attempts will facilitate timely, data-driven prevention efforts.

## Data Availability

The data that support the findings of this study are available from the corresponding author upon reasonable request due to privacy concerns.
